# Reproductive Counseling and Care in Cystic Fibrosis: A Multidisciplinary Approach for a New Therapeutic Era

**DOI:** 10.3390/life13071545

**Published:** 2023-07-12

**Authors:** Julie McGlynn, Joan K. DeCelie-Germana, Catherine Kier, Elinor Langfelder-Schwind

**Affiliations:** 1 Gynecology and Reproductive Sciences, Department of Obstetrics, Yale School of Medicine, New Haven, CT 06510, USA; julie.mcglynn@yale.edu; 2 Zucker School of Medicine at Northwell Division of Pediatric Pulmonary, and Cystic Fibrosis Center, Cohen Children’s Medical Center, New Hyde Park, NY 11040, USA; jgermana@northwell.edu; 3 Renaissance School of Medicine at Stony Brook, Department of Pediatrics, Stony Brook, NY 11794, USA; catherine.kier@stonybrookmedicine.edu; 4 The Cystic Fibrosis Center, Department of Pulmonary Medicine, Lenox Hill Hospital, Northwell Health, New York, NY 10075, USA

**Keywords:** cystic fibrosis, family planning, pregnancy, fertility, genetic counseling, highly effective modulators

## Abstract

With the advent of highly effective modulator therapies, many people with cystic fibrosis (CF) are living longer, healthier lives. Pregnancy rates for women with CF more than doubled between 2019 and 2021, reflecting increases in both planned and unplanned pregnancies. For men with CF, CF-associated infertility can be mitigated with assistive reproductive technology, yet patient knowledge of these challenges and options is variable. Preconception and prenatal counseling for individuals with CF and for parents of children with CF who wish to expand their families requires nuanced discussions to promote informed reproductive decisions, drawing from a combination of standard practice recommendations and CF-specific assessments. This review article synthesizes the current literature and practice recommendations regarding reproductive counseling and care in CF, outlining the role of genetic counseling, carrier screening, teratogen counseling, in vitro fertilization and pre-implantation genetic diagnosis, and careful assessment and management of cystic fibrosis-related diabetes when present. Via a multidisciplinary, patient-centered approach, clinicians can support adults with CF and parents of children with CF as they make informed reproductive decisions and embark on family planning.

## 1. Background and Significance

Cystic fibrosis (CF) is an inherited condition that affects the lungs, pancreas, and other organs. Variants in the cystic fibrosis transmembrane conductance regulator gene (*CFTR*) disrupt the production of the CFTR protein, which functions as an ion channel in the apical membrane of epithelial cells [1]. Over 2000 *CFTR* variants have been described, and more than 700 have been characterized as CF-causing [2]. Advances in the treatment and management of CF have led to significant improvements in survival and quality of life for children and adults over the last 20 years. According to the United States (US) Cystic Fibrosis Foundation Patient Registry (CFFPR), the median predicted age of survival for people with CF in the United States has increased from approximately 32 years in 2000 to more than 50 years in 2021 [3]. Most people with CF are adults and receive care at accredited CF care centers [3]. With consistent access to specialty care and new therapies, all individuals with CF are now expected to live well into adulthood.

Ongoing research and development of new treatments inform our optimism that survival and the outcomes for people with CF will continue to improve. The availability of new medications, highly effective CFTR modulators, starting with ivacaftor (IVA; brand name Kalydeco) in 2012 and elexacaftor/tezacaftor/ivacaftor (ETI; brand name Trikafta), in 2019, has led to improved lung function, attenuated disease progression, and improved nutrition. In the US, modulators are now available to over 90% of children and adults with CF over the age of 2 who have one or more responsive *CFTR* genetic variants, including the most common *CFTR* variant, F508del [4,5]; ivacaftor was recently approved for infants with responsive CFTR variants as young as one month of age [6]. However, despite widespread access in some countries, significant health disparities in CFTR modulator access exist, which can be attributed, in part, to the lower frequency of F508del in minoritized racial and ethnic groups [7]. Additionally, close attention to the benefits of nutritional support and management, such as the use of pancreatic enzyme replacement therapy and high-calorie, high-protein diets, have helped to prevent malnutrition and improve growth in people with CF [8]. These nutritional benefits begin right from birth, and the widespread implementation of CF newborn screening has enabled the early detection of infants with CF-related pancreatic insufficiency. Early detection has been especially critical because it spares children from developing failure to thrive and its developmental sequelae, which impacts pulmonary status as well [9].

**Figure 1:**
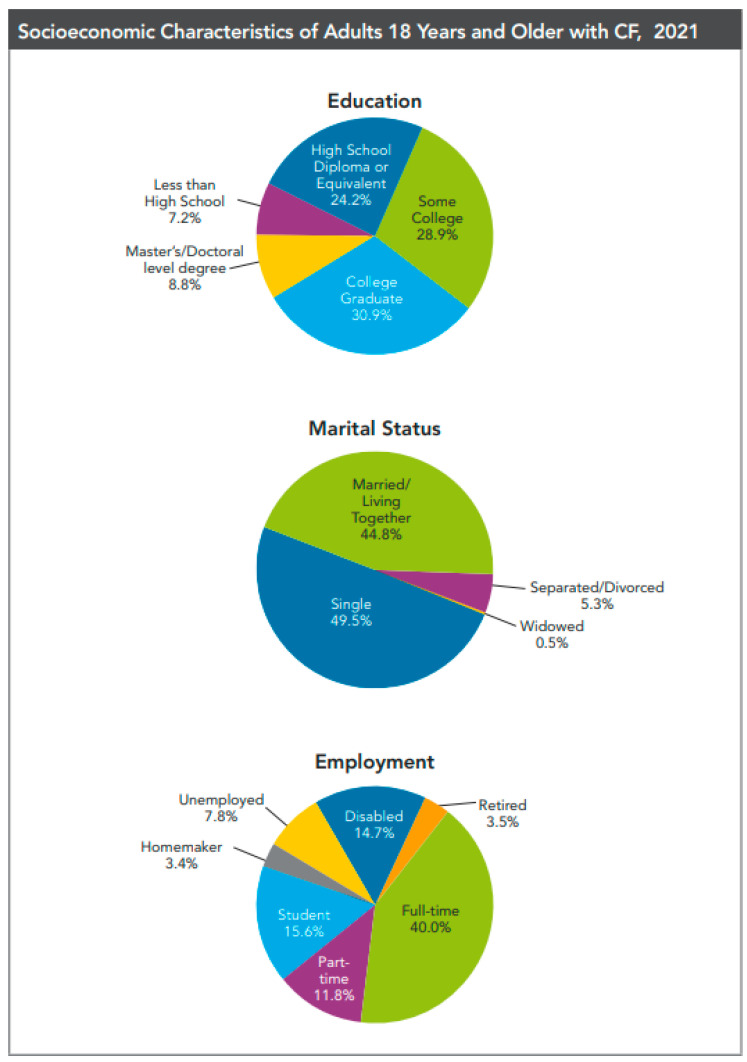
From the 2021 Cystic Fibrosis Foundation Patient Registry, reproduced with permission. In 2021, there were 32,100 individuals with CF in the Registry. Adults comprised 58.3 percent of the CF population (*n* = 18,714).

With an increased life expectancy and improvements in the quality of years lived, a growing percentage of the adult CF population can envision a future [10] where they have families of their own, and the discourse with parents of children with CF is changing as the prognosis improves. The CFFPR tracks relationship status as a quality-of-life measure for all people with CF, as well as pregnancy data for women. In 2021, CF care centers reported that 44.8% of adults with CF over 18 years of age were married or living with a partner; an additional 5.5% were divorced, separated, or widowed [3] Figure 1. At this time, data about whether a person with CF has children and how—by natural (spontaneous) conception, assisted reproductive technology, gestational surrogacy, adoption, or foster care—is not collected in the CFFPR; this is an area for future consideration. Heightened attention to investigating the sexual and reproductive health of and gender-specific changes in CF symptoms among individuals with CF is evidenced by a growing body of literature, spearheaded by the prolific CF Foundation-sponsored Sexual Health, Reproduction and Gender Research Working Group [11,12,13,14] and informed by a grass-roots, patient-directed effort to improve interactions about reproductive and sexual health between women with CF and their healthcare teams [15].

Individuals with CF have expressed a desire for frank conversations with trusted CF care providers about their reproductive goals and options, integrating a shared decision-making model into their CF care plans [16]. Balancing the dual roles of parent and patient, navigating prognostic uncertainty, anticipating the impact of future health decline, and the risk of early mortality on children [17] are sources of concern for both prospective and current mothers and fathers with CF [18] that require consideration, support, and empathy from the CF care team [19]. Additional areas for meaningful discussion include uncertainty about starting a family, uncertainty about future health, concerns about treatment adherence during and after pregnancy, the impact of pregnancy and childbirth on future health [20] and the chances of having a child with CF. These discussions about fertility and family building are layered conversations that optimally start in adolescence [21]. It is important for adolescents with CF to have a supportive healthcare team, including CF physicians, nurses, genetic counselors, social workers, and psychologists, who can provide accurate information, address concerns, and promote a holistic approach to reproductive health and sexuality. Open communication between healthcare providers, adolescents, and their families is crucial to ensure that a patient’s unique needs and questions are addressed in a respectful and age-appropriate manner [22].

Despite the growing interest in reproductive decision-making in CF, gaps in patient knowledge regarding fertility and reproductive options have been documented [23]. As such, appropriate counseling and tailored guidance are needed for individuals with CF and parents of children with CF to foster opportunities for open dialogue about their options for family building and to promote informed decision-making that is consistent with the individual’s and couple’s goals and values.

## 2. Genetic Counseling

Genetic counseling is the process of educating patients and families about the inherited basis of disease, integrating family history, risk assessments, curated information, and counseling to promote adaptation to the risk or diagnosis of a condition [24]. A trained, certified and/or licensed genetic counselor provides personalized information and support to couples in their family planning journey, assessing and addressing the risks of genetic disorders in a non-judgmental, non-directive manner that respects the patient or couple’s values and supports their decisions rather than persuading them to pursue a particular course of action [25]. Genetic counselors provide tailored information about genetics, inheritance patterns, and expected phenotypic spectrum for CF and other genetic conditions that are either present in the family history or commonly screened for in the preconception and prenatal period. Genetic counselors experienced in CF can help prospective parents appreciate the phenotypic spectrum of CF, ensuring that they understand that genotype is not entirely predictive of phenotype. Increasingly early access to CFTR modulator therapies in patients whose mutations and age render them eligible for treatment further substantiates that CF may impact future children differently from the family’s experience with their first child. In addition, genetic counselors can discuss options for pursuing parenthood, as well as the available screening and diagnostic testing, appropriate monitoring of an at-risk pregnancy, and recommendations for birth planning and neonatal care [26]. For example, genetic counselors can provide anticipatory guidance that a fetus or newborn known to have CF, or who is at an increased risk to have CF based on family history, should be evaluated by a pediatric CF care center for additional monitoring, discussion of a treatment plan, and diagnostic assessment [27]. Providing emotional support and facilitating connections to additional family building, obstetric, and postnatal resources complements and enhances the role of the other members of the care team [28]. Genetic counseling can improve patient knowledge and facilitate decision-making about family-building options [29].

Preconception and prenatal counseling for adults with CF and parents of children with CF require consideration of the benefits, limitations, and risks of the available testing options specific to CF as well as the standard practice recommendations for the general population. These include discussions of genetic carrier screening, residual reproductive risks of genetic disease if the carrier testing is negative, and pre- and postnatal diagnostic testing options [30]. For women with CF, additional attention is paid to the risk-benefit analysis of medications that enable them to remain healthy while pregnant and any potential impact they have on pregnancy and fetal development [31]. For all individuals with CF and couples with a child with CF, a critical aspect of preconceptual genetic counseling is to educate families about the variability of CF, the shifting prognosis given CFTR modulator therapies, and the fact that, although genotype-phenotype data may provide some guidance about the pulmonary and non-pulmonary features that an individual with CF may experience, there is significant intra- and inter-familial variability [32] as well as considerations related to having multiple close family members with CF [33].

## 3. Reproductive Decision-Making in Adults with CF

**Figure 2:**
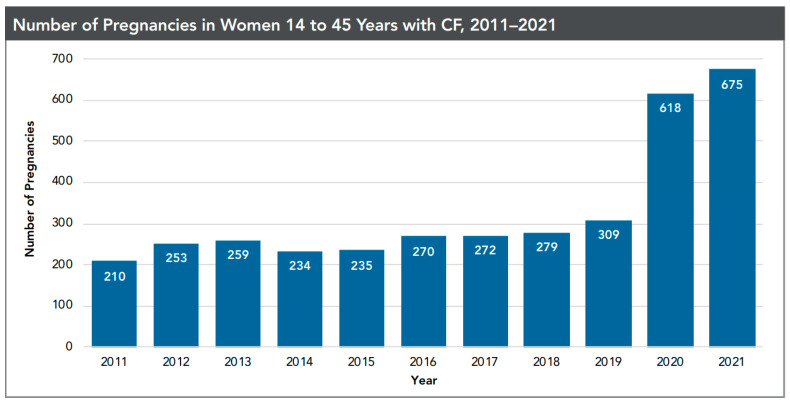
From the 2021 Cystic Fibrosis Foundation Patient Registry, reproduced with permission.

Biological females with CF are not expected to have anatomical barriers to conceiving spontaneously. However, before ETI, many women with CF had difficulty conceiving due to thickened cervical mucus, poor nutritional status, and decreased lung function [34,35]. Preconception planning should include a gynecological evaluation of fertility status to anticipate and explore options for conception, performed by a provider with experience with CF [36]. In a qualitative study of 25 women with CF who became parents, Bray et al. identified relationship status, health, finances, and personal preferences as key factors influencing both their decisions to pursue parenthood and to select a specific reproductive mechanism, such as natural/spontaneous conception, assisted reproduction, or surrogacy [37]. In 2019, prior to the availability of ETI, 310 pregnancies were reported to the CFFPR, reflective of an estimated five-fold increase in pregnancy rates among women with CF over the previous 20 years [38]. In 2021, more than double that number—675 pregnancies—were reported to CFFPR [3] (Figure 2). While this change may be attributable to an increase in the health and vitality of women with CF taking ETI, there was also a surprising increase in unplanned pregnancies, highlighting extrapulmonary implications of CFTR modulators [5] and the associated need for discussions about contraception and sexual activity in adolescents and women with CF [39]. This is especially important in light of the recent data regarding the increased medical risks for women with CF with unplanned pregnancies and their neonates [40].

The physiological demands of pregnancy are well-known to impact lung function in women [41]. Ideally, women with CF work closely with their CF clinical team to optimize their lung function before and during pregnancy [42], as successful pregnancy outcomes are known to correlate with nutritional and pulmonary statuses at conception. Women with CF and unplanned pregnancies and/or poor health status at conception have an increased frequency of pulmonary exacerbations, hospitalizations, and illness-related visits [43,44]. However, pregnancy does not appear to impact longer-term outcomes for women with CF [45], including women with moderate to severe lung disease [46]. Data regarding higher rates of premature delivery (<37 weeks) and primary C-sections have been reported [44,45,46] but not consistently. Pre-gestational diabetes is more common in mothers with CF, yet even after controlling for the impact of diabetes on pregnancy outcomes, one study found an increased likelihood of cardiac anomalies in infants born to women with CF [47], underscoring the need for fetal monitoring.

The prevalence of infertility in men with CF is estimated as 98% [48], primarily a result of obstructive azoospermia secondary to congenital bilateral absence of the vas deferens (CBAVD) [49]. One common CF-causing *CFTR* variant, c.3718-2477C > T (legacy name 3849 + 10Kb C > T), has an estimated prevalence of 1.8% among people with CF [3] and has been associated with male fertility [50]. Men with CF and CBAVD are expected to have viable sperm that can be retrieved [51], either directly from the epididymis via percutaneous epididymal sperm aspiration (PESA), from a small piece of testicular tissue via testicular sperm extraction (TESE) or by using direct microscopic examination of the testes via microdissection testicular sperm extraction (micro-TESE) [52]. After sperm retrieval, the partner’s egg (or donor egg) can be fertilized by using in vitro fertilization (IVF) with intracytoplasmic sperm injection (ICSI) [53]. In IVF with ICSI, a single sperm is injected directly into an egg, and the resulting embryo is then transferred to the partner’s uterus for implantation. Prior to embryo transfer, pre-implantation genetic testing (PGT) can be used in combination with IVF to screen the embryos for aneuploidy and single-gene genetic disorders, including CF, if the reproductive partner or ovum donor has also been identified as a CF carrier [54].

Consideration of the reproductive goals and the potential desire for fertility preservation also require discussion with individuals facing organ transplantation, although CF care providers may not feel equipped to engage fully in these conversations [55]. Egg retrieval and sperm retrieval and storage prior to transplant maximize the reproductive options for post-transplant pregnancy, as well as the possibility of surrogacy [56]. Fertility preservation is also relevant in other contexts, such as pre-oncologic and pre-gender affirming treatments, which are explored elsewhere [57]. Regardless of the clinical impetus, discussions regarding fertility preservation require a multidisciplinary approach that is timely, educational, and appropriate to the clinical situation [56].

## 4. Carrier Screening

Genetic carrier screening identifies the individuals/couples at increased risk of having children with specific genetic conditions, facilitates informed reproductive decision-making, and optimizes pre- and postnatal care. Prenatal carrier screening recommendations for an individual or couple are influenced by many factors, including ethnicity, family history, prior pregnancy history, insurance coverage, personal preferences and values, and local practice. Both patients and parents of children with autosomal recessive conditions, including CF, have been found to be supportive of genetic screening in principle but may not choose to utilize it for themselves [58,59]. At present, the American College of Obstetrics and Gynecologists (ACOG) recommends that, at a minimum, carrier screening for CF, spinal muscular atrophy, and certain inherited forms of anemia be offered to all individuals who are pregnant or planning to become pregnant [30]. Offering carrier screening for additional conditions may be recommended for individuals/couples who are at higher risk of carrying genetic variants for certain genetic disorders based on their ethnicity or personal or family history of a genetic disorder. Taking a detailed family history and documentation of genetic ancestry can inform risk assessment [60]; however, self-reported ethnicity is not a reliable predictor of genetic ancestry [61] and basing test offerings on ethnicity may further compound health disparities. Accordingly, The American College of Medical Genetics recommends a consistent and equitable approach whereby all patients are offered tiered carrier screening options, including testing for a broader range of predominantly autosomal recessive conditions and a limited number of X-linked conditions, such as fragile X syndrome and dystrophinopathies [62,63,64].

As with most genetic conditions, genetic carrier screening for CF can detect a significant percentage of, but not all, disease-causing variants. Individuals in the general population who receive a negative result have reduced, but not eliminated, their chance to be a CF carrier. Residual carrier risks are based upon the estimated test sensitivity and the baseline or the *a priori* chance of being a carrier and are typically less than half of one percent, meaning the risk is greatly reduced but greater than zero [65]. It is recommended that the labs offering CF carrier screenings assess a minimum panel of disease-causing *CFTR* variants; thus, when one member of a couple screens positive as a CF carrier or has CF, a limited genetic screening panel may be offered to the second partner, consistent with professional guidelines [30]. However, *CFTR* sequencing can provide additional carrier detection and permit a more refined reproductive risk assessment [66]. In counseling patients, it is critical to consider that, depending upon individual lab practices, genetic sequencing may also reveal variants of uncertain significance (VUS) and/or variants of varying clinical consequence (VCC), thereby complicating the discussion of reproductive risk with a broad phenotypic spectrum that includes mildly affected or asymptomatic individuals [67,68].

When both members of a couple are heterozygous carriers for an autosomal recessive condition, such as CF, with each pregnancy, there will be a 25% chance of having a child with the condition. If the partner of a person with CF is also a CF carrier, there is a one in two (50%) chance that each child will have CF. Targeted pre-implantation genetic testing (PGT) and prenatal testing are available for at-risk pregnancies in which the familial variants in the disease gene have been identified.

## 5. IVF with PGT

PGT is a screening test that can be performed on embryos created via IVF to genetically analyze the embryos for gene disorders and/or chromosome abnormalities prior to transfer into the uterus. After fertilizing the egg in a laboratory setting, one or more cells are removed from the trophectoderm and sent for genetic testing. Although PGT for CF is quite accurate and can significantly increase the chance of conceiving an unaffected pregnancy [69], embryos can be damaged in the process and may be unsuitable for transfer or fail to implant. Additionally, PGT may produce false-negative or false-positive results.

To reduce the error rate in PGT testing, it is recommended that original source DNA from a previously affected pregnancy or DNA from a carrier or affected parents be provided to the PGT lab [70]. Furthermore, while PGT is often pursued to avoid the risk of invasive prenatal diagnostic testing due to the chances of diagnostic error, confirmatory prenatal diagnosis is recommended after PGT [71]. As with all genetic tests, PGT does not detect all genetic abnormalities, only those tested. In the context of these limitations, PGT can help couples who are affected or carriers of genetic disorders, including CF, understand and refine or reduce the risk of passing on a genetic condition or chromosomal disorder to their children. Individuals and couples considering IVF with PGT should receive reproductive genetic counseling prior to embarking on this complex process.

Assisted reproductive technologies, with or without PGT, are expensive, and not all insurance plans cover the procedures, including most public insurance plans. Early referral to reproductive experts can help interested individuals/couples plan for the possibility of IVF with PGT by selecting an insurance plan that has IVF benefits, assisting with obtaining insurance pre-authorization, and/or identifying and accessing local resources, such as grants or other funding opportunities.

## 6. Prenatal Screening and Diagnostic Options

Once pregnancy is achieved, recommendations for prenatal genetic screening and diagnostic testing may again vary depending on factors that include the woman’s age, medical history, their partner’s medical history, and/or a family history of genetic disorders. Pregnant people are generally offered testing and screening options unrelated to cystic fibrosis, including nuchal translucency, serum marker, and/or cell-free DNA (cfDNA) screening to identify the risk of chromosomal abnormalities; second-trimester serum alpha-fetoprotein screening can be implemented to assess the risk for open neural tube defects, conducting ultrasounds to assess fetal growth and structural development, and prenatal diagnosis.

Prenatal diagnostic testing is available via chorionic villus sampling (CVS) or amniocentesis when both members of a couple are known to carry *CFTR* variants. In addition, emerging technologies, such as cell-free DNA screening for single-gene disorders, provide a non-invasive option for predicting the chance that a fetus will have CF [72]. However, cfDNA screening does not have the diagnostic accuracy afforded by CVS and amniocentesis, and false-positive and false-negative results can occur. Genetic counseling with a discussion of the benefits, limitations, and risks of the test options is recommended.

Preconception discussions with people with CF and parents of children with CF should maximize the ability of prospective parents to make informed decisions about their reproductive options and clearly convey that they will be supported by the medical team regardless of which approach they decide to pursue [25]. Decisions about having subsequent children after a child with CF are influenced by the existing family structure and parents’ lived experiences with CF [73]. If diagnostic testing reveals that a fetus will have CF, the couple faces options regarding pregnancy termination or continuation with expectant management. When a pregnancy is known to have CF, additional medical monitoring can be implemented in the form of serial prenatal ultrasounds to evaluate for echogenic bowel and the risk of meconium ileus and to facilitate birth planning and postnatal care [74,75]. Similarly, parents who opt to forego PGT or invasive prenatal testing assessments should be offered monitoring to identify and plan for any neonatal care needs that arise. Coordination with state newborn screening programs can confirm CF status for siblings via a genetic diagnosis, as sweat test practices for siblings vary across centers [76].

Overall, goals and preferences regarding reproductive planning and family building should be routinely assessed and supported, noting that they may change over time [73,77]. Non-biological family planning modalities, such as foster parenting, adoption, step-parenting, conception via donor gametes and surrogacy, are options for individuals with CF as well as for parents of children with CF. Prior to the development of techniques for sperm retrieval in men with CF, these options were likely to be utilized by men with CF to build their families [78]. The cost of some of these approaches can be prohibitive. Learning about the range of options for family building can facilitate decision-making, with or without changing a planned course of action [29].

## 7. Medication Uses during Pregnancy

Counseling about the potential teratogenic impact of CF medications in pregnancy is influenced by dose, timing, medication interactions, and the potential for any detrimental effects of discontinuing the medication. Fat-soluble vitamin (ADEK) dosing of a pregnant woman requires particular attention in the setting of increased dosing due to maternal deficiency [79]. Should a pulmonary exacerbation during pregnancy prompt consideration of antibiotics, a multidisciplinary approach is necessary. Consultation among the CF and obstetrics physicians, a CF pharmacist, a genetic counselor with expertise in teratogen assessment and counseling, and the adult patient can facilitate shared decision-making that addresses the health needs of the mother and minimizes teratogenic risks to the fetus [80].

While modulators have played a key role in promoting pregnancy in CF patients, the safety of ETI during pregnancy is under ongoing investigation. The MAYFLOWERS study (NCT04828382) was launched in September 2021 to track the experiences of women with CF who are conceiving while on ETI, to monitor the health of the pregnant women, the pregnancy, and to assess neonatal and early childhood outcomes of infants born to women on ETI in at least the first two years of life [13]. The study is expected to be completed at the end of 2025.

Animal studies of the individual components of ETI have shown some evidence of developmental toxicity, such as reduced fetal weight and skeletal abnormalities, as well as juvenile cataracts [81]. These risks are weighed against reports of a rapid decline in women with CF following discontinuation of CFTR modulators [82], coupled with early findings of healthy babies born to women who continued to take CFTR modulator therapy during pregnancy. More recent reports in the literature reveal cases of juvenile cataracts and elevated liver enzymes in babies exposed to ETI in utero and while breastfeeding [83].

In addition to these reported risks, two recent case reports suggest that ETI may be therapeutic when given prenatally in CF pregnancies. In one, an infant with CF whose mother remained on ETI throughout her pregnancy had a false-negative CF newborn screening result. Through coordinated postnatal communication and testing, the infant was subsequently diagnosed with CF and received appropriate treatment and monitoring [84]. A second case report involves a known CF carrier expecting her second child with CF. After dilated loops of bowel consistent with meconium ileus was noted on the fetal ultrasound, she began taking ETI. The resolution of the bowel distension was visualized after 27 days of treatment, and the baby was born free of intestinal obstruction [85]. The number of babies with CF born after experiencing in utero exposure to *CFTR* modulators may increase in the future as growing numbers of women with CF who are on modulators become pregnant. This nuanced and shifting landscape reinforces the importance of detailed discussions about the risks, benefits, limitations, and unknowns of remaining on ETI use during pregnancy [5]. Overall, it is critical that pregnancy in CF patients involves dedicated, specialized medical support so that individuals, in concert with their physicians, can make informed decisions about ETI during pregnancy.

## 8. CF-Related Diabetes

Cystic fibrosis-related diabetes (CFRD) is a common complication of CF and affects more than 50% of adults with CF. The Cystic Fibrosis Foundation guidelines include annual glucose and hemoglobin A1C levels and, starting at 10 years of age, an annual 2 h oral glucose tolerance testing [86]. Furthermore, as with type I or Type II diabetes, CFRD can have significant implications for pregnancy in women with CF and should be managed to assure good diabetic control in the months prior to conception and after completion of the pregnancy [87]. Additionally, women with CFRD may have more difficulty in controlling their blood sugar levels during pregnancy, which can lead to complications for both the mother and baby. In a French study, CFRD was associated with higher rates of assisted conception and increased risk of cesarean delivery [88], but not pre-eclampsia, preterm labor, or low birth weights, which has been reported elsewhere [89].

Regular discussion of family planning goals for women with CFRD is especially important to anticipate and address issues of glycemic control. Pre-conceptional supplementation with folic acid is recommended for all women but is especially critical for diabetic women to mitigate the increased risks of fetal neural tube defects associated with maternal diabetes [90]. Close monitoring of maternal and fetal health is needed to identify and manage any complications that may arise during pregnancy.

**Figure 3:**
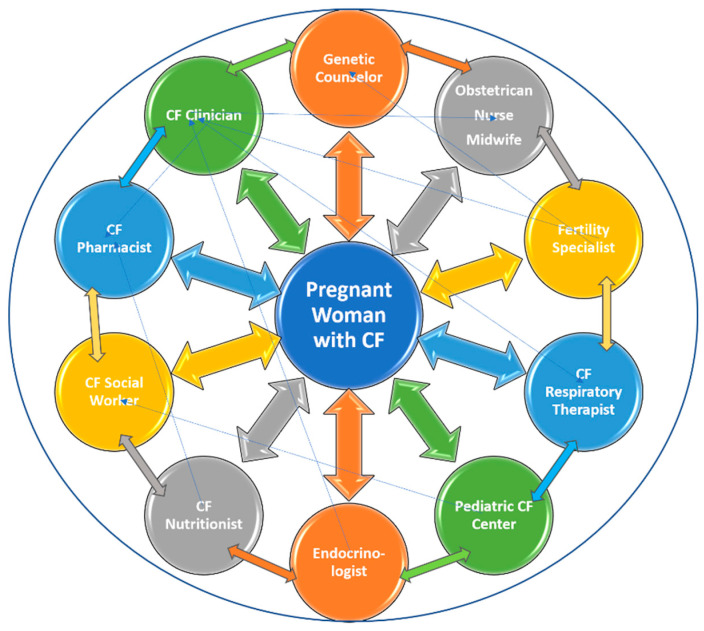
Clinical support and care team for a pregnant woman with CF. Pregnancy support system from CF team members and related specialties. Intersecting lines and arrows reflect the varying multidisciplinary care and communication needs.

Pregnant women with CF are expected to remain under the care of their local cystic fibrosis center, endocrinologist, and a high-risk obstetrical service to jointly monitor the woman’s pulmonary function, weight gain, glycemic control, blood pressure, and the fetus’s growth. Fetal monitoring must attend to the possibility of cystic fibrosis in the fetus and the effects of maternal medical conditions and medications. When a fetus is known or highly likely to have CF, delivery at a tertiary CF Center hospital should be considered, ensuring the appropriate support of the mother and baby and the coordination of maternal and infant care after delivery. A strong therapeutic relationship between the CF center and high-risk obstetrical service ensures the dual monitoring of the mother and fetus. Figure 3 illustrates the need for an interconnected, multidisciplinary approach to the care of a woman with CF.

## 9. Conclusions

The prognosis for individuals with cystic fibrosis has gradually evolved, with recent therapeutic advancements significantly improving overall health and outlook. Indeed, with the advent of highly effective modulator therapies, people with CF are living longer, healthier lives. Additionally, couples who are both CF carriers may now be more likely to continue CF-affected pregnancies, heartened by the availability of highly effective treatments. Preconception and prenatal counseling for adolescents and adults with CF, and for parents of children with CF who wish to expand their families, requires nuanced discussions. A timely, concerted discussion can promote informed reproductive decisions by drawing on a combination of standard practice recommendations and CF-specific assessments. These include genetic counseling regarding the risks, benefits, and limitations of carrier screening for CF and other available conditions, as well as the residual risks of having affected offspring after the screening. For women with CF, conversations to weigh the risks and benefits of medications that both enable them to remain healthy throughout the pregnancy and may potentially impact the pregnancy and fetal development are crucial. For men with CF, additional discussions of the etiology of male infertility and the options available to have biological children are recommended. Ideally, such discussions begin early, before reproductive concerns are pertinent and become more detailed in response to a patient’s questions and expressed reproductive intentions. For parents of children with CF, who have a 25% chance with each subsequent pregnancy of having another child with CF, preconception counseling enables parents to consider all available screening and diagnostic options, thereby maximizing their ability to make informed family planning decisions. A multidisciplinary approach to ongoing care and counseling about family planning goals and decisions, which often change over time, can benefit young adults with CF and parents of children with CF.

## Data Availability

Not applicable.
